# Association of renal resistive indices with kidney disease progression and mortality

**DOI:** 10.1186/s12882-023-03398-6

**Published:** 2023-11-28

**Authors:** Chloe Kharsa, Chadia Beaini, Dania Chelala, Mabel Aoun

**Affiliations:** 1 Faculty of Medicine, Saint Joseph University of Beirut, Beirut, Lebanon; 2 Department of Nephrology, Faculty of Medicine, Saint Joseph University of Beirut, Beirut, Lebanon

**Keywords:** Renal resistive index, Chronic Kidney Disease, Hemodialysis, Life style modifications, Mortality

## Abstract

**Background:**

Renal resistive indices (RRI) have been shown to predict the progression of kidney disease. This study aims to evaluate the association of RRI with mortality and dialysis initiation after adjustment to therapeutic and life style interventions.

**Methods:**

This is a retrospective study that included all chronic kidney disease patients followed for at least two years in three nephrology clinics between 2006 and 2019 and who had a RRI level in their files. Kaplan Meier and log rank test compared the survival of patients with normal versus high RRI. Cox regression analysis evaluated the association between RRI and death or dialysis initiation after adjustment to treatments and life style modifications.

**Results:**

A total of 192 patients were analyzed: 68 had RRI < 0.7 and 124 had RRI ≥ 0.7. Their mean age was 66.5 ± 13.1 years at first visit, 78.1% were males. There was a negative correlation between baseline eGFR and RRI (*p* < 0.001; Spearman correlation coefficient = -0.521). The survival was significantly better in patients with RRI < 0.7 with a Log Rank test < 0.001. The univariate cox regression analysis showed a significant association between RRI and mortality (HR = 1.08; 95%CI: 1.04–1.11; *p* < 0.001) that remained significant after adjustment to cardiovascular risk factors and interventions such as salt reduction, blood pressure control, statins and RAAS inhibitors (HR = 1.04; 95%CI: 1.00–1.08; *p* = 0.036). Cox regression analysis showed a significant association between RRI and dialysis initiation (HR = 1.06; 95%CI 1.01–1.10; *p* = 0.011).

**Conclusion:**

Our study revealed that patients with an elevated RRI ≥ 0.7 are at a higher risk of mortality after adjustment to medications and lifestyle modifications. RRI can, according to this study, be considered as an independent prognostic factor in CKD patients.

**Supplementary Information:**

The online version contains supplementary material available at 10.1186/s12882-023-03398-6.

## Introduction

Chronic kidney disease (CKD) is defined as an alteration of the kidney function and structure during more than three months [1, 2]. The prevalence of CKD patients is growing significantly affecting at least one in ten adults [3]. This can be explained by improvement in life expectancy, by aging of populations [4] and by the increase of risk factors of kidney diseases [5], such as obesity, hypertension [6] and diabetes [7]. CKD is currently a major global public health issue and it is one of the leading causes of death worldwide [8]. It is still a challenge to determine the prognostic markers of CKD and to evaluate whether these markers are independently associated with outcomes.

Kidney ultrasound (US) is the gold standard imaging to rule out urinary tract obstruction, to assess kidneys' size and corticomedullary differentiation [9]. When kidney US is coupled to a pulsed wave spectral Doppler, vascular velocities in the renal main artery and intra-renal arteries can be evaluated. Doppler US is usually prescribed to diagnose renal artery stenosis. The variation in blood flow velocity with time evaluated by Doppler US makes it possible to calculate the renal resistive index (RRI) [10]. RRI is defined as the maximum blood flow velocity in systole minus the minimum blood flow velocity at the end of diastole over peak systolic velocity. RRI was initially used to evaluate arterial stiffness [11]. The normal value of RRI in an adult varies between 0.47 and 0.7 with a difference that does not exceed 5 to 8% between the two kidneys [12]. RRI directly reflects the vascular impedance, which results from the interaction between pulsatility and vascular compliance [13]. Thus, any condition that decreases vascular compliance and increases pulse pressure induces an increase in RRI, for example advanced age, smoking, hypertension, atherosclerosis and CKD.

RRI has been demonstrated by several researchers as a renal and cardiovascular prognostic marker [14–18]. In hypertensive patients without prior cardiovascular disease, RRI predicted the renal outcome and overall survival [19, 20]. In patients with diabetes, RRI predicted the occurrence of diabetic nephropathy [14, 15]. Elevated RRI seem to reflect the kidney microvasculature and the degree of tubulointerstitial disease [21]. High RRI predicted a resistance to steroid therapy in glomerulonephritis [22]. In transplant patients, the prognostic value of RRI > 0.7 or > 0.8 was also extensively studied [16, 23, 24]. Pulse pressure was found as an independent predictor of high RRI in transplant patients [25] and RRI < 0.8 at 3 months after transplantation was associated with better kidney prognosis [26]. RRI is also useful in the diagnosis of acute kidney injury (AKI) and a cut-off of 0.7 was predictive of post-operative AKI and related-mortality [17, 27]. Despite all these studies highlighting the prognostic role of RRI, physicians are still not using doppler US for this purpose.

Therefore, this study aims to evaluate the association of high RRI with renal outcome and mortality in CKD patients and to assess whether this association persists after adjustment to therapeutic and life style modifications.

## Materials and methods

### Study design, context and participants

This is a multi-center retrospective study that included CKD patients who consulted at three Lebanese nephrology clinics for the first time between February 2006 and December 2019. Nephrologists following these patients are affiliated to the Faculty of Medicine of the Saint-Joseph University of Beirut.

### Eligibility criteria

Patients were included if they were older than 18 years, if they had a renal Doppler US with a RRI level in their file and if they were followed for at least two years. Single (solitary) kidney patients, kidney transplant recipients, patients diagnosed with polycystic kidney disease or renal artery stenosis were excluded.

### Data collection

Data collection was conducted between April and September 2022. Baseline data collected from patients' medical files included demographics, date of first visit, number of visits, cardiovascular (CV) risk factors such as hypertension, diabetes, smoking, dyslipidemia, obesity, history of coronary artery disease (CAD), heart failure and/or strokes, laboratory values at the first visit (T1) such as serum creatinine, glomerular filtration rate (GFR) estimated by the 2012 CKD-EPI equation, urine albumin over creatinine ratio (ACR), HbA1c level. RRI levels were collected once from reports of Doppler US at T1 or between the first and second visit. If RRI levels of the two kidneys were different, we recorded the average of both levels.

Follow-up data included the number of visits, date of last visit, laboratory values of serum creatinine, eGFR, ACR and HbA1c at last visit (T2), chronic medications namely statin, proton pump inhibitor (PPI), calcium channel blocker (CCB), renin–angiotensin–aldosterone system (RAAS) inhibitor (Angiotensin-converting enzyme (ACE) inhibitor or angiotensin-receptor blocker), beta-blocker, thiazide diuretic and occurrence of any new cardiovascular event such as coronary event, heart failure, or stroke.

### Definitions

CKD is defined in this study based on the KDIGO classification taking into consideration GFR categories and urine albumin to creatinine ratio (ACR) [1]. GFR category is estimated by the 2012 CKD-EPI equation. Stage 1 is an estimated GFR (eGFR) ≥ 90 ml/min/ 1.73 m2 with ACR ≥ 30 mg/g or any other marker of kidney damage, stage 2 is eGFR 60–89 with ACR ≥ 30 mg/g or any other marker of kidney damage, stage 3a is eGFR 45–59, stage 3b is eGFR 30–44, stage 4 is eGFR and stage 5 is eGFR < 15 ml/min/ 1.73 m2.

### Life style modifications/ interventions

We collected whether blood pressure was controlled, defined as < 140/90 at the last visit (T2), whether HbA1c was reduced between first and last visit. Other recorded life style modifications were smoking cessation, weight loss and compliance with a low sodium diet. Weight loss was defined as any weight loss above 2 Kgs between T1 and T2. Patients were considered as compliant to low salt diet based on their statement, and/or their caregiver's confirmation and/or low 24-h urinary salt less than 5 g per day when available. The compliance to medications was assessed by the count of boxes that the patients bring to the clinic in order to renew the unified prescription and to get reimbursed by the national social security fund or the military funds of the country, that constitute 80% of all patients' coverage. For the remaining 20%, we assessed patients' compliance by the renewed prescriptions.

### Outcomes

Two outcomes were recorded: death and initiation of dialysis. The time before death and/or dialysis has been determined, as well as the cause of mortality. Another outcome assessed was the three-point major cardiovascular event (3P-MACE) including non-fatal myocardial infarction, non-fatal stroke or cardiovascular death.

### Measurements

Radiologists affiliated to the three clinics were well-trained at performing RRI measurement using Doppler US. RRI is defined as the maximum blood flow velocity in systole minus the minimum blood flow velocity at the end of diastole over peak systolic velocity.

The laboratory biological parameters including creatinine level, ACR and HbA1c were measured using standard techniques in the three hospitals’ labs.

### Statistical analysis

Statistical analyses were performed using the Statistical Package for the Social Sciences, Version 24.0 (SPSS Inc.-IBM corp., Armonk, NY, USA). Continuous data were reported as mean and standard deviation (SD) if normally distributed and as median and interquartile (IQR) if skewed. Categorical data were reported as numbers and percentages. Missing data was estimated at 7.8% for compliance to salt reduction, 6% for smoking cessation, 11.5% for weight loss and 49.5% for ACR (T2). ACR at T2 was removed from the analysis. Little's MCAR test showed that compliance to salt reduction, smoking cessation and weight loss were missing completely at random. We performed a multiple imputation regression model to replace the missing values. The imputed data was used to analyze the multivariable cox regression model and the results were similar to the original model. Mann Whitney test, independent t-test, Chi Square test were used to compare two groups of RRI levels. Spearman rho correlation evaluated the correlation between two continuous variables. The receiver operating curve (ROC) analysis was used to assess the predictive value of RRI for dialysis and death. Kaplan Meier survival analysis and log rank test evaluated the difference in survival between patients with normal and high RRIs. Cox regression analysis assessed the factors associated with death and the association between RRI and dialysis. A Cox proportional hazards regression analysis was performed to assess factors associated with death, dialysis and 3P-MACE; the first model included all cardiovascular risk factors, the second, third and fourth models added to Model 1 renal factors, therapeutic and preventive interventions. *P-*value < 0.05 was considered as statistically significant.

## Results

### Flowchart diagram

**Fig. 1:**
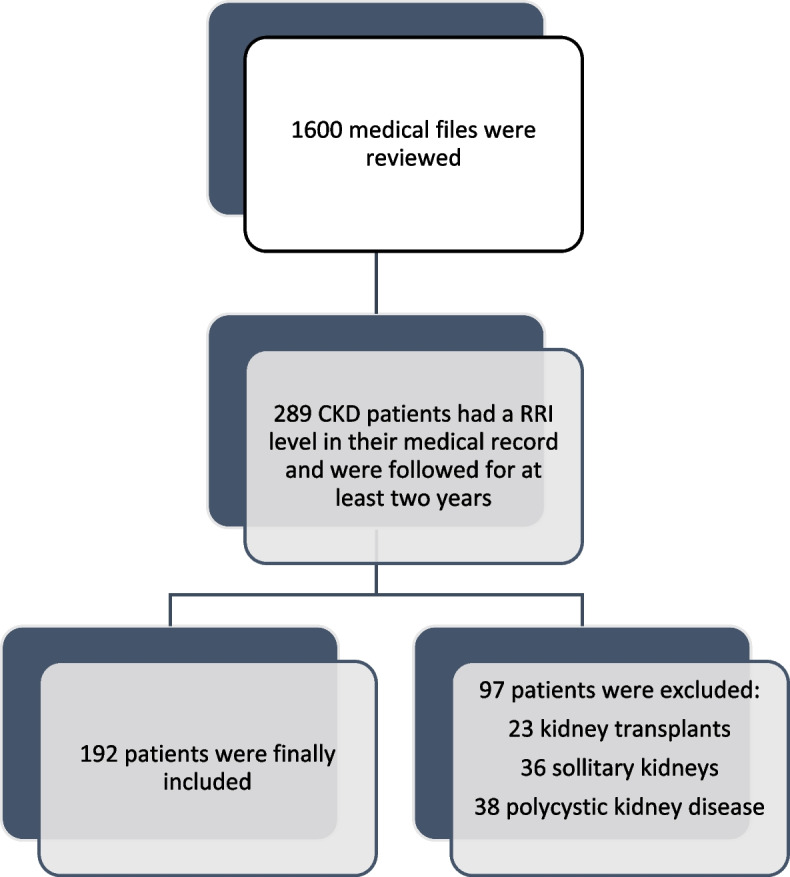
Flow diagram of patients' inclusion

Among the 1600 medical files reviewed in three different clinics, 289 patients responded to the inclusion criteria (the major reason for non-inclusion was the absence of RRI level in the medical file). Were excluded: 23 kidney transplants, 36 solitary kidneys and 38 polycystic kidney disease patients. Finally, a total of 192 CKD patients responded to inclusion and exclusion criteria (Fig. 1).

### General characteristics

Out of the 192 patients analyzed, 124 had a RRI ≥ 0.7. Table 1 summarizes their baseline characteristics. Their mean age was 66.5 ± 13.1 years at first visit, 78.1% were males, 95.8% of them were hypertensive. Their mean follow-up was 73.4 ± 41.5 months.

**Table 1:** General characteristics of patients divided into normal and high RRI

Variable	Total *N* = 192	Patients with RRI < 0.7 *N* = 68	Patients with RRI ≥ 0.7 *N* = 124	*P*
**Demographics**				
**Age at first consultation, y** **Mean ± SD**	66.5 ± 13.1	58.7 ± 12.7	70.7 ± 11.2	< 0.001
**Sex (M/F), n(%)**	150/42 (78.1/21.9)	54/11 (79.4/20.6)	96/28 (77.4/22.6)	0.75
**Smoking, n(%)**	83 (43.2)	32 (47.1)	51(41.1)	0.33
**Obesity, n(%)**	65 (33.9)	27 (39.7)	38 (30.6)	0.17
**Cardiovascular risk factors**				
**Hypertension, n(%)**	184 (95.8)	64 (94.1)	120 (96.8)	0.38
**Diabetes, n(%)**	93 (48.4)	23 (33.8)	70 (56.5)	0.003
**Dyslipidemia, n(%)**	152 (79.2)	48 (70.6)	104 (83.9)	0.03
**History of CAD, n(%)**	56 (29.2)	13 (19.2)	43 (34.6)	0.02
**History of stroke, n(%)**	14 (7.3)	3 (4.4)	11 (8.9)	0.26
**History of heart failure, n(%)**	16 (8.3)	3 (4.4)	13 (10.5)	0.15
**Cause of CKD, n(%)**				
• Diabetic nephropathy	93 (48.4)	23 (33.8)	70 (56.5)	0.01
• Cardiorenal syndrome	3 (1.6)	0	3 (2.4)	
• Glomerular disease	2 (1)	1 (1.5)	1 (0.8)	
• Undetermined or tubulointerstitial nephritis or nephrosclerosis	94 (49)	44 (64.7)	50 (40.3)	
**Laboratory parameters T1**				
**Serum creatinine T1, mg/dL** **Median (IQR)**	1.6 (1.2–2.2)	1.3 (0.9–1.6)	1.8 (1.4–2.6)	< 0.001
**eGFR, mL/min/1.73m2 T1**	40 (28–57.8)	59 (40.8–81.8)	35 (22.3–45)	< 0.001
**Stage of CKD T1, n(%)**				
• Stage 1	16 (8.3)	13 (19.1)	3 (2.4)	< 0.001
• Stage 2	31 (16.1)	21 (30.9)	10 (8.1)	
• Stage 3a	33 (17.2)	14 (20.6)	19 (15.3)	
• Stage 3b	58 (30.2)	14 (20.6)	44 (35.5)	
• Stage 4	46 (24)	6 (8.8)	40 (32.3)	
• Stage 5	8 (4.2)	0	8 (6.5)	
**ACR T1, mg/g** **Median (IQR)**	194 (29–1000)	66 (12.5–263)	500 (47.5–1650)	< 0.001
**HbA1c T1 (if diabetes)** **Median (IQR)**	6.9 (6–7.9)	7.3 (6.4–9.6)	7.1 (6.5–8.2)	0.63
**RRI, T1** **Median (IQR)**	0.73 (0.65–0.80)	0.6 (0.6-0.65)	0.8 (0.73-0.85)	< 0.001

**Fig. 2:**
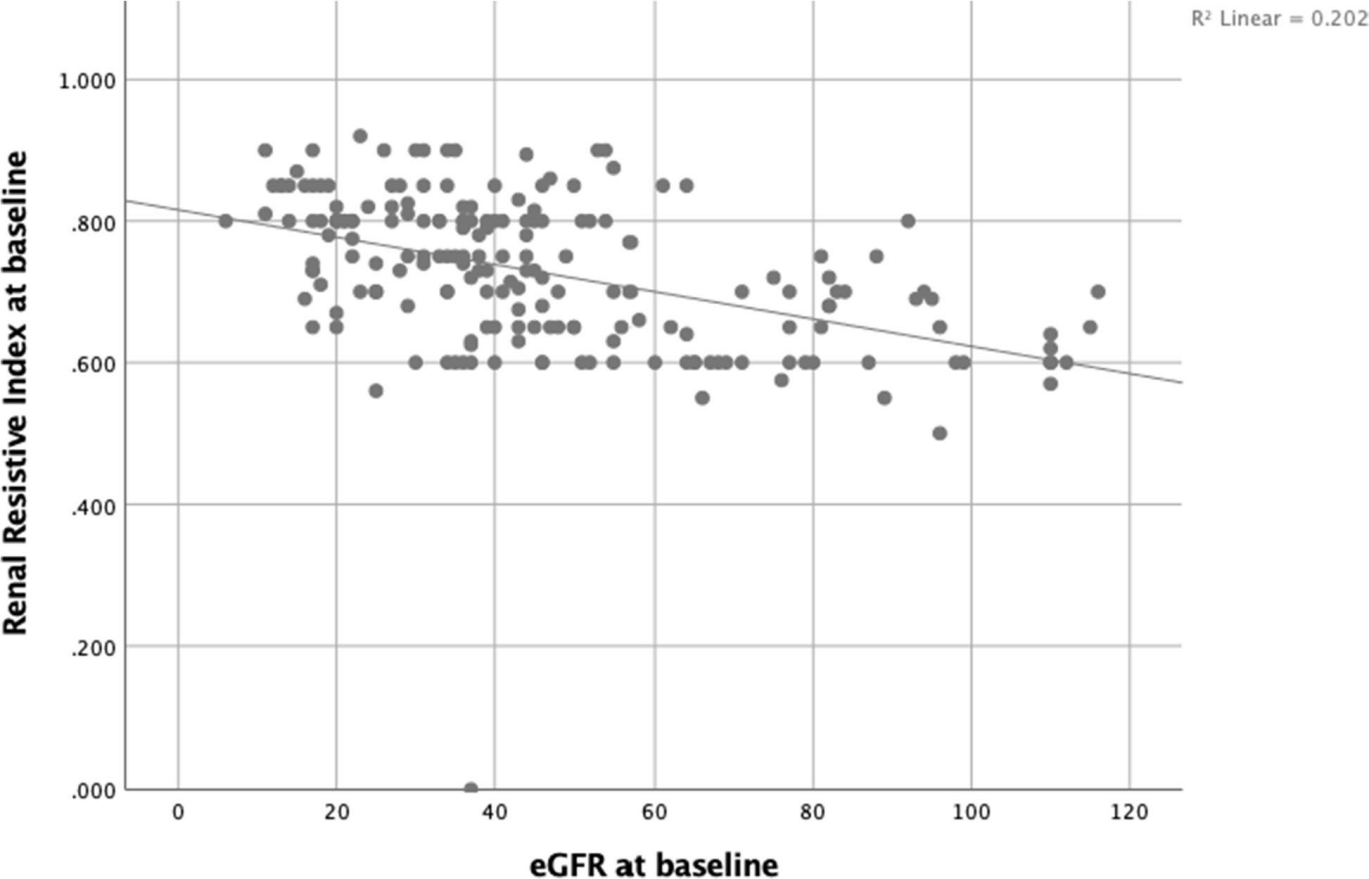
Correlation between RRI and eGFR at baseline

The Spearman correlation coefficient showed a negative correlation between baseline eGFR and RRI (coefficient = -0.521, *P* < 0.001) (Fig. 2).

### Medications and life style modifications

Percentages of patients treated with calcium channel blockers, proton pump inhibitors and beta blockers were higher in the group with a RRI ≥ 0.7 compared to the group with normal RRI.

Over the course of consultations, cardiovascular risk factors were less controlled in patients with a RRI ≥ 0.7. Moreover, only 48% managed their blood pressure and 36% followed a low-sodium diet in contrast to patients with a normal RRI, of whom 71% controlled their BP and 54% followed a low-salt diet (Table 2).

**Table 2:** Follow-up: treatment and lifestyle modifications

Variable	Total *N* = 192	Patients with RRI < 0.7 *N* = 68	Patients with RRI ≥ 0.7 *N* = 124	*P*
Treatment				
Statin, n(%)	124 (64.6)	44 (64.7)	80 (64.5)	0.979
ACE inhibitor, n(%)	26 (13.5)	12 (17.6)	14 (11.3)	0.203
Angiotensin Receptor Blocker, n(%)	105 (54.7)	39 (57.4)	66 (53.2)	0.509
Thiazide, n(%)	30 (15.6)	8 (11.8)	22 (17.7)	0.293
CCB, n(%)	121 (63)	37 (54.4)	84 (67.7)	0.087
PPI, n(%)	48 (25)	11 (16.2)	37 (29.8)	0.041
Antiaggregant agent, n(%)	86 (44.8)	26 (38.2)	60 (48.4)	0.204
Beta-blocker, n(%)	113 (58.9)	29 (42.6)	84 (67.7)	0.001
Number of antihypertensive molecules, Mean ± SD	2.07 ± 0.96	1.87 ± 0.89	2.18 ± 0.99	0.027
Follow-up, interventions/lifestyle modifications				
Duration of follow-up, months Mean ± SD	73.4 ± 41.5	85.8 ± 46.6	66.6 ± 36.8	0.002
Number of consultations during follow-up (before dialysis), months Median (IQR)	5 (3–10.8)	4 (2–6.8)	6 (3–12)	0.002
Control of hypertension < 140/90, n(%)	108 (56.3)	48 (70.6)	60 (48.4)	0.006
HbA1c reduction in patients with diabetes, n(%)	46 out of 93 (49.5)	14 out of 23 (61)	33 out of 70 (47.1)	0.460
Weight loss, n(%)	60 (31.3)	23 (33.8)	37 (29.8)	0.490
Smoking cessation among smokers, n(%)	13 out of 83 (15.7)	8 out of 32 (25)	6 out of 51 (11.8)	0.100
Compliance to salt reduction, n(%)	82 (42.7)	37 (54.4)	45 (36.3)	0.009

### Complications during follow-up and outcomes

**Fig. 3:**
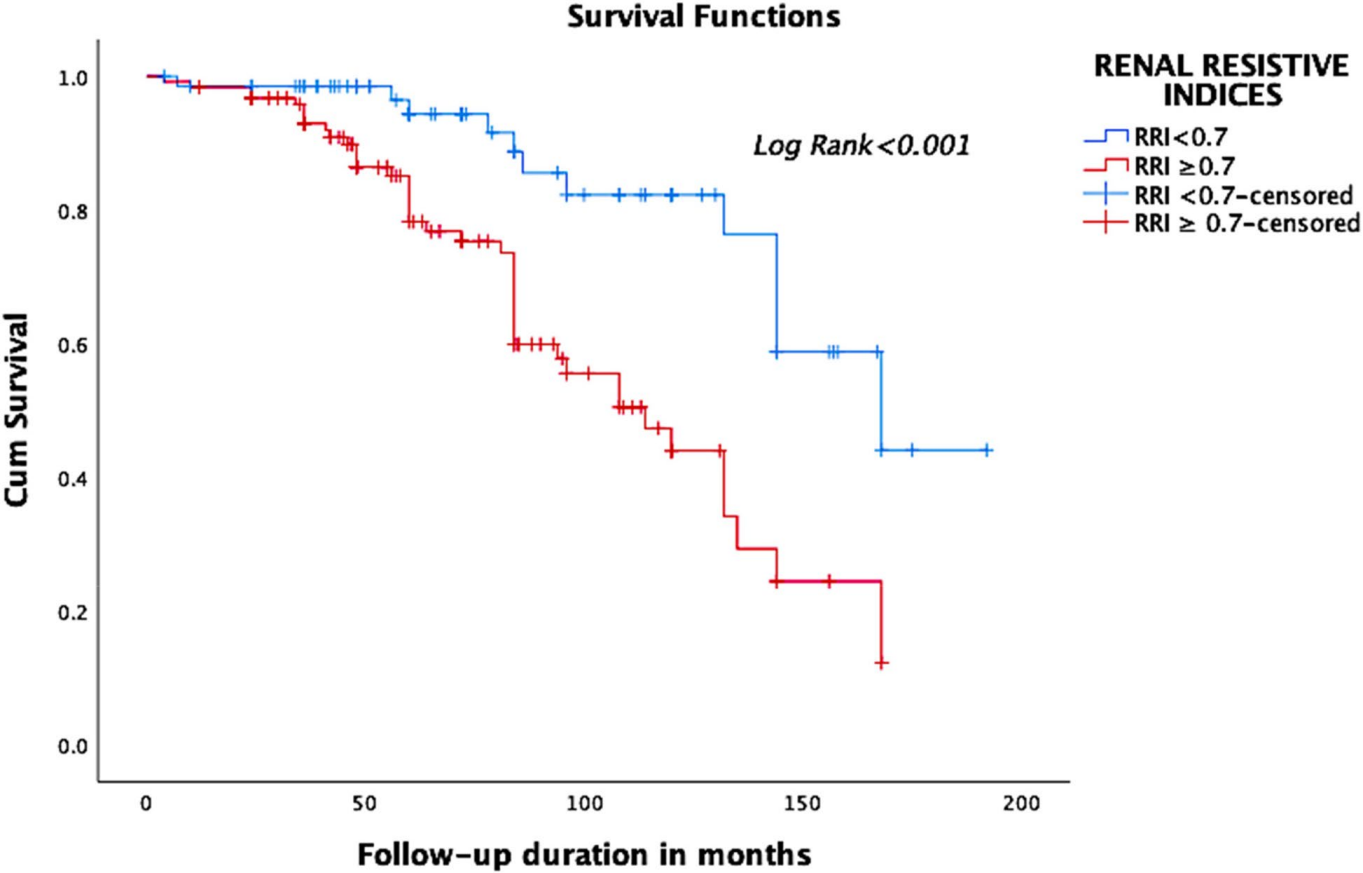
Survival curve of two groups of RRIs

Patients with high RRI ≥ 0.7 had higher rates of doubling of serum creatinine, of initiation dialysis and of death (Table 3). The survival curve obtained during the Kaplan Meier analysis of the two groups shows a better life expectancy in patients with a normal renal resistive index with Log Rank < 0.001 (Fig. 3).

**Table 3:** Follow-up: complications, renal outcomes and death

**Variable**	**Total** ***N*** ** = 192**	**Patients with RRI < 0.7** ***N*** ** = 68**	**Patients with RRI ≥ 0.7** ***N*** ** = 124**	***P***
**Complications during follow-up**				
**Coronary event, n(%)**	31 (16.1)	13 (19.1)	18 (14.5)	0.313
**Stroke, n(%)**	8 (4.2)	3 (4.4)	5 (4)	0.851
**Heart failure, n(%)**	12 (6.3)	4 (5.9)	8 (6.5)	0.915
**Outcomes**				
**Serum creatinine, mg/dL T2** **Median (IQR)**	1.9 (1.1–4.5)	1.2 (0.9–1.9)	2.5 (1.5–5.9)	< 0.001
**eGFR, mL/min/1.73m2 T2** **Median (IQR)**	33.5 (10.3–60.8)	61 (31–87)	22.5 (8–42.8)	< 0.001
**Doubling of serum creatinine, n(%)**	42 (21.9)	6 (8.8)	36 (29)	0.001
**Dialysis, n(%)**	40 (20.8)	8 (11.8)	32 (25.8)	0.022
**Duration of follow-up until dialysis, months** **Mean ± SD**	38.2 ± 25.4	63.5 ± 28.1	31.8 (20.6)	< 0.001
**Death, n(%)**	54 (28.1)	12 (17.6)	42 (33.9)	0.017
**Cause of death, n(%)**				
• Cardiac cause	26 (13.5)	6 (8.8)	20 (16.1)	0.02
• Stroke	1 (0.5)	0	1 (0.8)	
• Cancer	13 (6.8)	5 (7.4)	8 (6.5)	
• Infection	3 (1.5)	0	3 (2.4)	
• COVID	8 (4.2)	0	8 (6.5)	
• Bleeding	1 (0.5)	0	1 (0.8)	
• Cirrhosis	1 (0.5)	1 (1.5)	0	
• Trauma	1 (0.5)	0	1 (0.8)

**Fig. 4:**
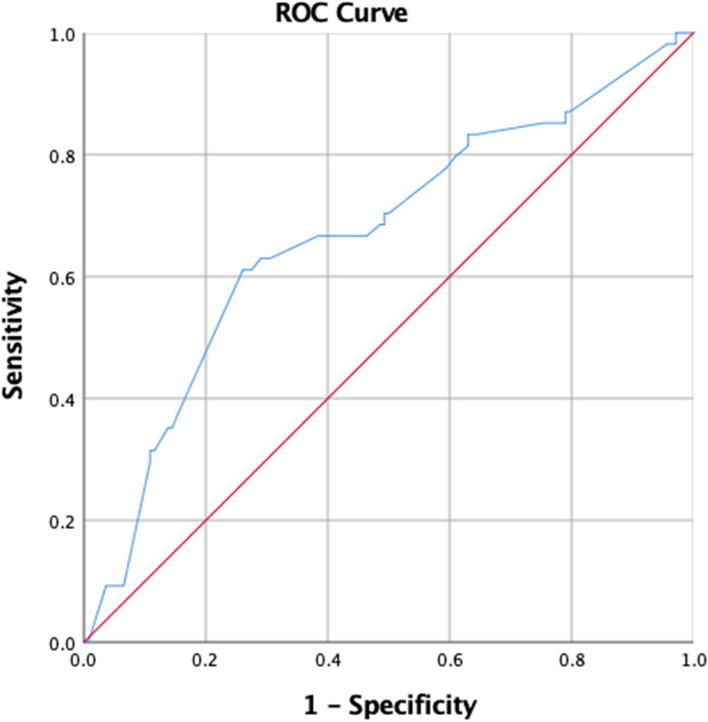
Area under the ROC curve of RRI predicting death

**Fig. 5:**
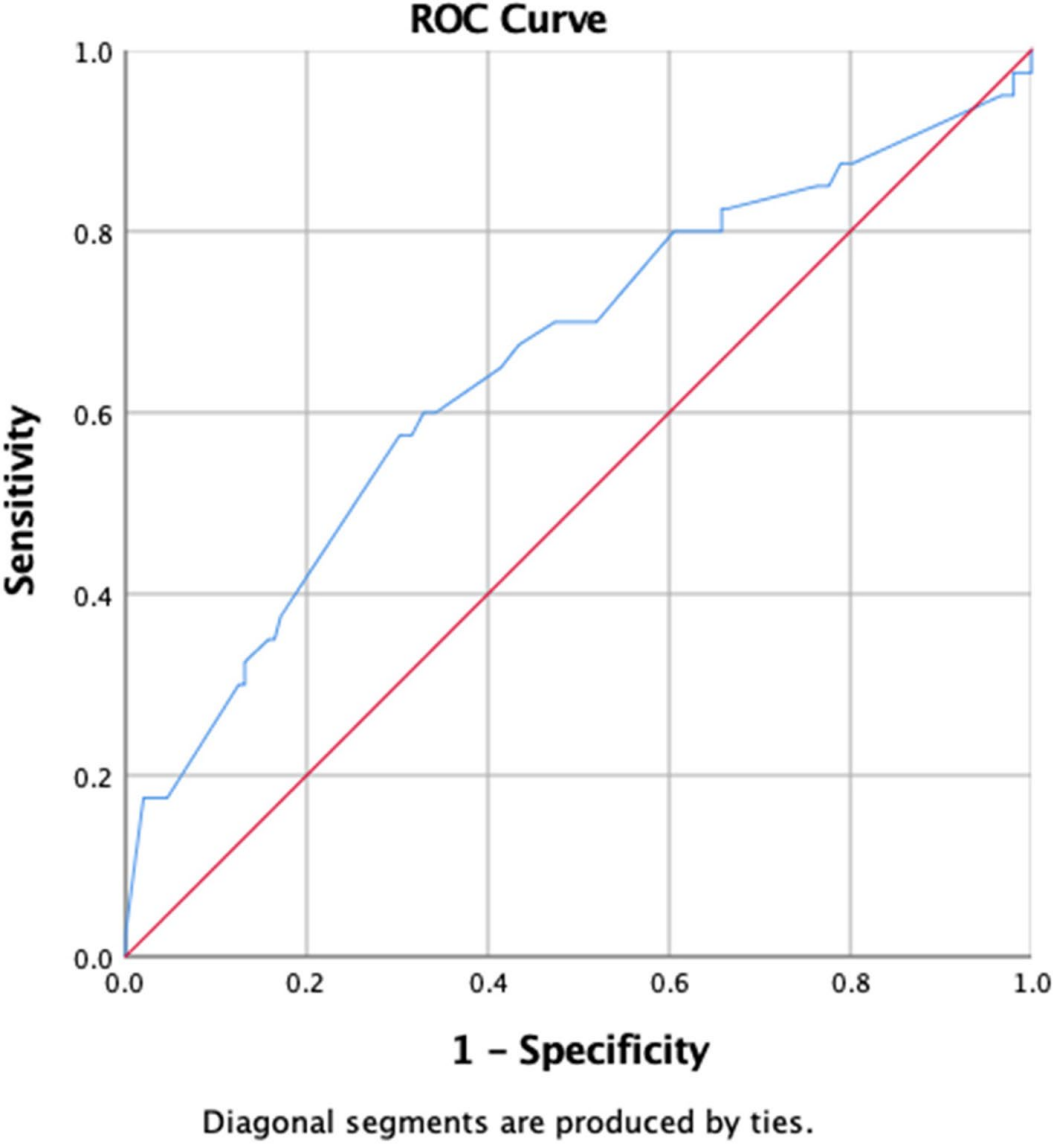
Area under the ROC curve of RRI predicting dialysis

The area under ROC curve for RRI predicting death is 0.667 (Fig. 4), and the area under ROC curve for RRI predicting dialysis is 0.654 (Fig. 5).

### Factors associated with dialysis occurrence

Cox regression analysis showed that baseline elevated RRI, lower eGFR, high levels of albuminuria were risk factors associated with dialysis occurrence (Table 4). RAAS inhibitors, compliance to salt reduction and control of BP to less than 140/90 were all protective factors (Table 4). The number of visits over follow-up duration was significantly associated with dialysis (Table 4).

**Table 4:** Cox regression analysis of factors associated with dialysis (univariate analysis)

	***Univariate analysis***		
	HR	95% Confidence Interval	*P*
**RRI**	1.05	1.02–1.09	0.003
**Age**	1.01	0.98–1.03	0.762
**Sex (** *Ref: Male)*	0.60	0.25–1.44	0.255
**Diabetes**	1.95	1.03–3.67	0.040
**Dyslipidemia**	1.65	0.69–3.93	0.259
**Obesity**	0.95	0.49–1.85	0.880
**CAD**	1.65	0.87–3.13	0.127
**Heart failure**	1.76	0.69–4.49	0.238
**Stroke**	0.83	0.20–3.45	0.797
**eGFR, mL/min**	0.90	0.88–0.93	< 0.001
**ACR, g/g**	1.51	1.36–1.69	< 0.001
**RAAS inhibitors**	0.49	0.27–0.93	0.028
**Statins**	0.76	0.41–1.44	0.404
**Beta-blockers**	1.94	0.98–3.83	0.056
**Antiaggregants**	1.56	0.83–2.91	0.167
**PPIs**	1.23	0.63–2.42	0.548
**BP control < 140/90**	0.44	0.23–0.82	0.010
**Compliance to salt reduction**	0.22	0.09–0.50	< 0.001
**HbA1c reduction in patients with diabetes**	0.73	0.32–1.65	0.442
**Smoking cessation in smokers**	0.56	0.13–2.46	0.443
**Number of visits over follow-up duration**	7.89	4.63, 13.43	< 0.001

*Hypertension was not assessed because the vast majority had hypertension; RRI was multiplied by 100*

After adjusting to cardiovascular risk factors, RRI remained an independent risk factor associated with dialysis (Table 5 and Table S1). RRI was no more associated with dialysis after adjustment to eGFR, ACR, treatments and lifestyle modifications (Table 5 and Table S1).

**Table 5:** Cox proportional hazards models associated with dialysis

	***Multivariable analysis***		
	**HR**	**95% Confidence Interval**	***P***
**MODEL 1 (CV risk factors)**			
**RRI**	1.06	1.01–1.10	0.012
**MODEL 2 (CV risk factors, renal factors, treatment and lifestyle modifications)**			
**RRI**	0.99	0.96–1.04	0.830
**MODEL 3 (Subgroup of patients with diabetes)**			
**RRI**	1.09	0.99–1.21	0.081

*Model 1 adjusted to: Age, sex, dyslipidemia, obesity, CAD, diabetes, heart failure, history of stroke, number of antihypertensives*

*Model 2 included Model 1* + *eGFR, ACR, RAASi, statins, beta-blockers, antiaggregants, compliance to salt reduction, control of BP* < *140/90*

*Model 3 included Model 2* + *HbA1c reduction*

### Factors associated with mortality

Cox regression analysis showed a significant association between RRI and death (HR: 1.08; CI: 1.04–1.11; *P* < 0.001) (Table 6). Age, CAD, heart failure, low eGFR, beta-blockers were also factors associated with excess death whereas taking a RAAS inhibitor was protective (Table 6).

**Table 6:** Cox regression analysis of factors associated with death (univariate analysis)

	***Univariate analysis***		
	HR	95% Confidence Interval	*P*
**RRI**	1.08	1.04–1.11	< 0.001
**Age**	1.04	1.02–1.07	0.001
**Sex (** *Ref: Male)*	1.60	0.89–2.87	0.118
**Diabetes**	1.40	0.82–2.39	0.224
**Dyslipidemia**	0.77	0.41–1.44	0.410
**Obesity**	0.75	0.42–1.35	0.334
**CAD**	2.19	1.26–3.80	0.008
**Heart failure**	2.28	1.11–4.68	0.025
**Stroke**	0.54	0.07–3.96	0.546
**eGFR, mL/min**	0.97	0.96–0.98	< 0.001
**ACR, g/g**	1.13	0.99–1.28	0.058
**RAAS inhibitors**	0.41	0.24–0.69	0.001
**Statins**	0.63	0.37–1.08	0.093
**Beta-blockers**	1.93	1.08–3.46	0.027
**Antiaggregants**	1.46	0.85–2.51	0.176
**PPIs**	0.69	0.36–1.34	0.273
**Number of antihypertensives**	0.86	0.64–1.16	0.325
**BP control < 140/90**	0.90	0.53–1.54	0.703
**HbA1c reduction in patients with diabetes**	0.99	0.49–2.01	0.997
**Salt reduction**	0.68	0.38–1.23	0.207
**Smoking cessation in smokers**	0.34	0.08–1.43	0.141
**Number of consultations**	0.99	0.96, 1.02	0.394

After adjusting to cardiovascular risk factors, renal factors, treatments and lifestyle modifications, RRI remained an independent risk factor associated with death (Table 7 and Table S2).

**Table 7:** Cox proportional hazards models associated with death

	***Multivariable analysis***		
	**HR**	**95% Confidence Interval**	***P***
**MODEL 1 (CV risk factors)**			
**RRI**	1.07	1.03–1.11	< 0.001
**MODEL 2 (CV Risk factors and renal factors)**			
**RRI**	1.04	0.99–1.07	0.030
**MODEL 3 (CV risk factors, renal factors, treatment and lifestyle modifications)**			
**RRI**	1.05	1.00–1.08	0.020
**MODEL 4 (Subgroup of patients with diabetes)**			
**RRI**	1.07	0.98–1.18	0.119

*Model 1 adjusted to: Age, sex, dyslipidemia, obesity, CAD, diabetes, heart failure, history of stroke, number of antihypertensives*

*Model 2 included Model 1* + *eGFR, ACR,*

*Model 3 included Model 2* + *RAASi, statins, beta-blockers, antiaggregants, compliance to salt reduction, control of BP* < *140/90*

*Model 3 included Model 3* + *HbA1c reduction*

### Factors associated with 3P-MACE

Logistic regression analysis showed a significant association between RRI and 3P-MACE (HR: 1.05; CI:1.02–1.09; *P* = 0.001), that remained significant after adjustment to CV risk factors (HR = 1.04; CI:1.01–1.07; *P* = 0.039). This association was not statistically significant after adding eGFR and albuminuria to demographics and CV risk factors (HR = 1.03; CI:0.99–1.06; *P* = 0.130) and after further adding of treatment and lifestyle modifications (HR = 1.04; CI:0.99–1.08; *P* = 0.070).

## Discussion

This study confirms RRI as an independent factor associated with death in patients with CKD. According to our results, an increase of 0.01 in RRI increases the death by 7% after a mean follow-up of 73 months. This association is sustained after adjustment to cardiovascular risk factors, kidney function, ACR, therapeutic and lifestyle interventions. Our results concur with Toledo et al. who analyzed 1962 patients with CKD stages 3 and 4 and found that RRI > 0.7 was associated with higher mortality [18]. Similarly, Romano et al. followed 131 patients with a mean age of 76 years, for a median of 7.5 years and found that patients with RRI ≥ 0.80 had a faster kidney function loss and higher mortality [28], and similar to our results, their AUROCs of RRI for predicting mortality and progression of renal disease were 0.67 and 0.66 respectively [28]. Leodori et al. followed also 122 patients with systemic sclerosis and different levels of eGFR and demonstrated that RRI is an independent predictor of mortality [29].

Another important finding in our study is the strong correlation between RRI and eGFR at baseline. This has been previously described by several researchers. Kosaki et al*.* recently demonstrated that patients with CKD have an increased intrarenal pulsatility and an elevated RRI [30]. Sistani et al. showed significant association between RRI and GFR and albuminuria among 100 patients with diabetic nephropathy [31]. Bigé et al. evaluated RRI levels in 58 patients two days prior to kidney biopsy and found a strong correlation between RRI and interstitial fibrosis as well as accelerated kidney function decline independent of baseline eGFR [32]. This strong association between eGFR and RRI suggest that the outcomes driven by RRI depend on the kidney function. This might be true for dialysis in our study but not for mortality. In fact, Toledo et al. showed also that the association of RRI with mortality remained significant after adjustment to the kidney function [18]. Regarding the dialysis outcome, our study revealed a significant association between RRI and the progression to dialysis. However, in contrast to mortality, the association of RRI and dialysis was no longer statistically significant after adjusting to different comorbidities. On the contrary, Parolini et al. followed 86 patients with CKD for 2–11 years and found out that RRI ≥ 0.7 was an independent risk factor for the progression to renal failure, independent of initial eGFR [33]. Barone et al. recently showed that a high RRI was a predictive factor for deterioration of renal function after coronary angiography [34].

One of the main objectives of this study was to evaluate the RRI value in predicting dialysis or mortality, after adjusting to nephroprotective treatment and lifestyle modifications. This was an indirect way to assess whether RRI prognostic value could be modified after implementing appropriate therapeutic and preventive interventions. In fact, very few studies assessed the direct impact of treatment on RRI. Yamaguchi et al. studied 100 CKD patients treated with RAAS inhibitors and who had two measures of RRI [35]. They found that RAAS inhibitors could lower RRI levels [35]. Leoncini et al. compared a small sample of patients treated with lisinopril versus nifedipine; they found more significant reduction in RRI under lisinopril [36]. Our analysis showed that RAAS inhibitors had a protective impact both on death and dialysis, however RRI remained an independent prognostic factor for mortality after adjusting to RAAS inhibitors. The retrospective design of our study does not allow us to draw strong conclusions but it is a call for future interventional studies to assess the long-term effect of RAAS inhibitors on RRI. Many studies have already proven the cardiovascular protection of RAAS inhibitors in chronic kidney disease patients but they have not stratified their patients into low and high RRI [37].

The other factor that emerged as protective against dialysis in the subgroup of patients with diabetes was the compliance to salt reduction. In fact, many studies have shown the beneficial effect of dietary sodium restriction on kidney function and the positive synergistic effect of RAAS blockade combined to salt reduction [38]. Unfortunately, the definition of compliance to salt reduction in our study was not always based on the 24-h urine sodium but also on patients and caregivers' statements which could be subject to bias. This is another call for interventional trials to assess RRI levels before and after compliance to salt reduction, specifically in patients with diabetes.

### Limitations and strengths

To the best of our knowledge, this is the first study analyzing the prognostic character of RRI in CKD patients after adjustment to therapeutic and preventive interventions. Although some might argue that RRI can be operator-dependent, all RRI measurements were performed by well-trained radiologists who are referees in kidney Doppler US in our country. Despite this fact, we admit that some slight variations might occur due to different operators. On the other hand, some limitations should be noted. The major limitation remains in the retrospective nature of our study and the absence of Doppler US after implementation of treatment and life style modifications. A second limitation is the possible bias in patient selection; it is not clear why some patients underwent renal Doppler US while others did not. This limitation makes our findings generalizable only for patients who underwent RRI measurement. The reasons behind RRI measurement in included patients are most of all the fact that these patients got their renal ultrasound by one of the radiologists who measure systematically RRI, the second less common cause is the decline in renal function following RAASi. The third limitation is related to the definition of compliance to salt reduction that was not homogenous in all cases.

## Conclusion

The renal resistive index is an important diagnostic and prognostic element to consider in the renal and cardiovascular evaluation and management of chronic kidney disease. It appears as a very sensitive prognostic marker, predicting progression to an advanced stage of renal failure or death. Despite lifestyle changes and compliance to therapeutic interventions, RRI seems to be an independent prognostic marker of mortality and a diagnostic tool reflecting the severity of renal disease.

### Supplementary Information

**Additional file 1:** **Table S1**. Cox regression analysis of factors associated with dialysis (multivariable analysis). **Table S2.** Cox regression analysis of factors associated with death (multivariable analysis). 

## Data Availability

The dataset supporting the conclusions of this article is included within the article (and its additional file).

## Abbreviations

RRI: Renal resistive index or indices
CKD: Chronic kidney disease
GFR: Glomerular filtration rate
CV: Cardiovascular
CAD: Coronary artery disease
ACR: Albumin to creatinine ratio
PPI: Proton pump inhibitor
CCB: Calcium channel blocker
RAAS: Renin–angiotensin–aldosterone system
ACE: Angiotensin-converting enzyme
