# Supplementary material for: Association of renal resistive indices with kidney disease progression and mortality
Source: BMC Nephrol. 2023 Nov 28;24:348. doi: 10.1186/s12882-023-03398-6 (PMC10685556; doi:10.1186/s12882-023-03398-6)
Supplement: Supplementary file 1 — Additional file 1: Table S1. Cox regression analysis of factors associated with dialysis (multivariable analysis). Table S2. Cox regression analysis of factors associated with death (multivariable analysis). [file 12882_2023_3398_MOESM1_ESM.docx]

**Table S1**. Cox regression analysis of factors associated with dialysis (multivariable analysis)

|  | *Multivariable analysis* | | |
| --- | --- | --- | --- |
|  | **HR** | **95% Confidence Interval** | ***P*** |
| MODEL 1 (CV risk factors) | | | |
| RRI | 1.06 | 1.01-1.10 | 0.012 |
| Age | 0.98 | 0.95-1.01 | 0.100 |
| Sex (*Ref: Male)* | 0.56 | 0.22-1.38 | 0.205 |
| Dyslipidemia | 1.29 | 0.53-3.18 | 0.572 |
| Obesity | 0.77 | 0.38-1.56 | 0.471 |
| CAD | 1.28 | 0.61-2.70 | 0.515 |
| Diabetes | 1.39 | 0.70-2.74 | 0.346 |
| Heart failure | 1.20 | 0.43-3.38 | 0.728 |
| History of stroke | 0.54 | 0.13-2.35 | 0.414 |
| Number of antihypertensives | 1.09 | 0.76-1.57 | 0.619 |
| MODEL 2 (CV risk factors, renal factors, treatment and lifestyle modifications) | | | |
| RRI | 0.99 | 0.96-1.04 | 0.830 |
| Age | 1.01 | 0.97-1.04 | 0.782 |
| Sex (*Ref: Male)* | 0.18 | 0.06-0.57 | 0.004 |
| Obesity | 0.34 | 0.12-0.95 | 0.041 |
| Dyslipidemia | 1.16 | 0.33-4.14 | 0.815 |
| CAD | 0.67 | 0.26-1.76 | 0.421 |
| Diabetes | 1.00 | 0.38-2.61 | 0.996 |
| Heart failure | 1.99 | 0.61-6.48 | 0.255 |
| History of stroke | 1.01 | 0.19-5.32 | 0.994 |
| Number of antihypertensives | 0.76 | 0.33-1.75 | 0.513 |
| eGFR, mL/min | 0.90 | 0.86-0.94 | <0.001 |
| ACR, g/g | 1.57 | 1.27-1.92 | <0.001 |
| RAAS inhibitors | 0.83 | 0.27-2.51 | 0.740 |
| Statins | 0.48 | 0.18-1.23 | 0.126 |
| Beta-blockers | 2.25 | 0.69-7.22 | 0.175 |
| Antiaggregants | 0.33 | 0.14-0.82 | 0.017 |
| Compliance to salt reduction | 0.39 | 0.14-1.15 | 0.089 |
| Control of BP<140-90 | 0.66 | 0.23-1.89 | 0.441 |
| MODEL 3 (Subgroup of patients with diabetes) | | | |
| RRI | 1.09 | 0.99-1.21 | 0.081 |
| Age | 1.05 | 0.95-1.14 | 0.343 |
| Sex (*Ref: Male)* | 0.08 | 0.01-0.64 | 0.017 |
| Obesity | 0.95 | 0.24-3.79 | 0.947 |
| Dyslipidemia | 1.35 | 0.02-76.82 | 0.884 |
| CAD | 0.94 | 0.12-7.46 | 0.955 |
| Heart failure | 117.7 | 5.7-2432.2 | 0.002 |
| History of stroke | 1.11 | 0.09-12.73 | 0.933 |
| Number of antihypertensives | 0.59 | 0.04-8.53 | 0.703 |
| eGFR, mL/min | 0.90 | 0.82-0.99 | 0.037 |
| ACR, g/g | 1.72 | 1.26-2.35 | 0.001 |
| RAAS inhibitors | 0.06 | 0.01-1.42 | 0.082 |
| Statins | 0.84 | 0.20-3.52 | 0.813 |
| Beta-blockers | 8.73 | 0.71-107.9 | 0.091 |
| Antiaggregants | 0.19 | 0.04-0.99 | 0.050 |
| Compliance to salt reduction | 0.02 | 0.00-0.40 | 0.012 |
| Control of BP<140-90 | 1.47 | 0.23-9.24 | 0.683 |
| HbA1c reduction | 6.94 | 1.24-38.74 | 0.027 |

**Table S2.** Cox regression analysis of factors associated with death (multivariable analysis)

|  | *Multivariable analysis* | | |
| --- | --- | --- | --- |
|  | **HR** | **95% Confidence Interval** | ***P*** |
| MODEL 1 (CV risk factors) | | | |
| RRI | 1.07 | 1.03-1.11 | <0.001 |
| Age | 1.03 | 0.99-1.06 | 0.081 |
| Sex (*Ref: Male)* | 2.60 | 1.35-5.02 | 0.004 |
| Obesity | 0.67 | 0.32-1.39 | 0.280 |
| Dyslipidemia | 0.89 | 0.48-1.69 | 0.741 |
| CAD | 2.84 | 1.43-5.65 | 0.003 |
| Diabetes | 0.96 | 0.53-1.73 | 0.894 |
| Heart failure | 1.35 | 0.59-3.03 | 0.474 |
| History of stroke | 0.50 | 0.07-3.78 | 0.503 |
| Number of antihypertensives | 0.68 | 0.49-0.95 | 0.023 |
| MODEL 2 (CV Risk factors and renal factors) | | | |
| RRI | 1.04 | 0.99-1.07 | 0.030 |
| Age | 1.03 | 0.99-1.06 | 0.087 |
| Sex (*Ref: Male)* | 3.34 | 1.29-5.70 | 0.002 |
| Obesity | 0.92 | 0.42-1.62 | 0.822 |
| Dyslipidemia | 0.88 | 0.37-2.09 | 0.771 |
| CAD | 3.03 | 1.23-5.04 | 0.004 |
| Diabetes | 0.94 | 0.53-1.90 | 0.841 |
| Heart failure | 1.18 | 0.49-2.62 | 0.710 |
| History of stroke | 0.59 | 0.07-4.37 | 0.626 |
| Number of antihypertensives | 0.64 | 0.43-0.93 | 0.021 |
| eGFR, mL/min | 0.99 | 0.97-1.00 | 0.086 |
| ACR, g/g | 0.97 | 0.82-1.16 | 0.916 |
| MODEL 3 (CV risk factors, renal factors, treatment and lifestyle modifications) | | | |
| RRI | 1.05 | 1.00-1.08 | 0.020 |
| Age | 1.03 | 0.99-1.07 | 0.099 |
| Sex (*Ref: Male)* | 3.19 | 1.29-7.31 | 0.010 |
| Obesity | 0.58 | 0.29-1.61 | 0.200 |
| Dyslipidemia | 1.31 | 0.49-3.44 | 0.590 |
| CAD | 3.19 | 1.53-8.91 | 0.010 |
| Diabetes | 1.28 | 0.63-2.54 | 0.493 |
| Heart failure | 1.95 | 0.77-4.84 | 0.159 |
| History of stroke | 0.70 | 0.08-5.26 | 0.743 |
| Number of antihypertensives | 0.45 | 0.23-0.86 | 0.016 |
| eGFR, mL/min | 0.99 | 0.97-1.01 | 0.378 |
| ACR, g/g | 1.05 | 0.83-1.23 | 0.629 |
| RAAS inhibitors | 1.34 | 0.28-1.35 | 0.553 |
| Statins | 0.83 | 0.36-1.74 | 0.678 |
| Beta-blockers | 3.49 | 0.62-3.22 | 0.032 |
| Antiaggregants | 0.76 | 0.36-1.87 | 0.524 |
| Compliance to salt reduction | 1.01 | 0.43-2.21 | 0.989 |
| Control of BP<140-90 | 2.04 | 0.92-4.97 | 0.109 |
| MODEL 4 (Subgroup of patients with diabetes) | | | |
| RRI | 1.07 | 0.98-1.18 | 0.119 |
| Age | 1.03 | 0.95-1.12 | 0.445 |
| Sex (*Ref: Male)* | 3.90 | 0.63-24.19 | 0.143 |
| Obesity | 0.47 | 0.09-2.34 | 0.357 |
| Dyslipidemia | 0.88 | 0.06-12.47 | 0.925 |
| CAD | 10.70 | 1.55-74.09 | 0.016 |
| Heart failure | 0.35 | 0.02-5.05 | 0.438 |
| History of stroke | 0.33 | 0.01-12.37 | 0.551 |
| Number of antihypertensives | 0.08 | 0.01-0.56 | 0.012 |
| eGFR, mL/min | 0.98 | 0.93-1.03 | 0.405 |
| ACR, g/g | 1.19 | 0.90-1.56 | 0.210 |
| RAAS inhibitors | 2.92 | 0.42-20.24 | 0.278 |
| Statins | 1.26 | 0.24-6.54 | 0.781 |
| Beta-blockers | 6.45 | 0.83-49.96 | 0.075 |
| Antiaggregants | 0.74 | 0.18-3.08 | 0.675 |
| Compliance to salt reduction | 1.04 | 0.14-7.65 | 0.971 |
| Control of BP<140-90 | 2.13 | 0.33-13.67 | 0.427 |
| HbA1c reduction | 8.05 | 1.04-62.59 | 0.046 |
